# Editorial: Brain circuitry and neuropsychiatric outcomes in COVID-19

**DOI:** 10.3389/fpsyt.2026.1946794

**Published:** 2026-08-24

**Authors:** Zora Kikinis, Bianca Besteher, Stefano Pallanti

**Affiliations:** 1 Brigham Women’s Hospital, Department of Psychiatry, Harvard Medical School, Boston, MA, United States; 2 Department of Psychiatry and Psychotherapy, Jena University Hospital, Jena, Germany; 3 German Center for Mental Health, Das Deutsche Zentrum für Psychische Gesundheit (DZPG), Jena, Germany; 4 Center for Intervention and Research on Adaptive and Maladaptive Brain Circuits Underlying Mental Health (C-I-R-C), Site Halle-Jena-Magdeburg, Jena, Germany; 5 Department of Psychiatry and Behavioral Science, Albert Einstein College of Medicine, Bronx, NY, United States; 6 Istituto di Neuroscienze, Florence, Italy

**Keywords:** COVID - 19, neurocognition, neuroimaging (anatomic), post-acute sequelae of SARS-CoV-2 infection (PASC), psychological burden, vulnerable populations

## Introduction

The global footprint of the coronavirus disease 2019 (COVID-19) pandemic extends far beyond acute respiratory distress, presenting a lingering challenge to mental health and neurological stability. Months after acute SARS-CoV-2 infection, a substantial subset of individuals continue to struggle with persistent, disabling manifestations, a complex syndrome widely categorized as Post-Acute Sequelae of SARS-CoV-2 infection (PASC), Post-COVID-19 Condition (PCC), or “Long COVID”. Among these long-term symptoms, neuropsychiatric deficits—including cognitive impairment, profound fatigue, anxiety, depression, and executive dysfunction—are particularly prevalent and debilitating. This Research Topic compiles seven articles that collectively clarify the structural, psychological, and physiological mechanisms driving^1^ these post-viral syndromes.

## Neuroimaging and brain circuitry alterations

Central to understanding the persistent cognitive deficits in Long COVID is mapping structural alterations within the human brain. Advanced neuroimaging reveals complex modifications in cortical macrostructure and neural circuitry.

While early literature frequently highlighted cortical atrophy, recent evidence reveals a more nuanced structural landscape. A structural magnetic resonance imaging (MRI) study by Joshi et al. demonstrated localized cortical hypertrophy in Long COVID patients. Specifically, significant increases in cortical thickness were observed in the cortical limbic network and executive control hubs, including the caudal anterior, isthmus, and posterior cingulate gyrus, alongside the rostral middle frontal gyrus. Higher gray matter volume (GMV) was also found in the posterior and isthmus cingulate. These structural enlargements correlate with clinical dementia ratings and anxiety scores, pointing toward compensatory structural remodeling or underlying neuroinflammatory swelling.

Furthermore, voxel-based morphometry (VBM) investigations by Toepffer et al. highlight that cognitive impairment in Long COVID follows distinct, sex-specific gray matter patterns. In females with cognitive impairment (Montreal Cognitive Assessment [MoCA] score < 26), GMV alterations are localized primarily within anterior frontal, limbic, and diencephalic regions. Conversely, affected males present with more widespread neocortical alterations involving a higher number of affected clusters across the parietal, occipital, and motor cortices. These diverging regional vulnerabilities strongly suggest distinct underlying neurobiological mechanisms between sexes, highlighting the necessity for personalized, sex-stratified treatment strategies.

At a systems level, these macroscopic structural shifts are deeply intertwined with complex brain-immune interactions. As detailed in a review by Kikinis et al., modern immunobiology views systemic immunity as an integrated network directly shaping autonomic, limbic, and cognitive behaviors. Histopathological and neuroimaging analyses validate that the inflammatory cascade triggered by SARS-CoV-2 infection compromises the structural architecture of the brain’s immune-responsive networks. This persistent neuroimmune disruption provides a biological framework explaining how peripheral viral infections translate into long-term neuropsychiatric deficits.

## Quantifying and characterizing the neurocognitive profile

The term “brain fog” describes the heterogeneous cognitive symptoms reported by PCC patients, including mental fatigue, reduced cognitive clarity, and disrupted focus. Standardizing the assessment of this subjective burden is vital for clinical translation and person-centered rehabilitation.

A new scale, the Brain Fog Scale (BFS), validated by Loizidou et al., retains a stable three-factor structure: (1) impaired cognitive acuity, (2) inattentiveness, and (3) mental exhaustion. Statistical evaluations demonstrate that individuals diagnosed with PCC exhibit significantly higher total brain fog symptoms compared to those without PCC. Furthermore, “impaired cognition” is the dominant driving factor separating post-COVID cohorts from controls, affirming the scale’s clinical utility.

When cognitive symptoms are mapped using domain-specific batteries like the Oxford Cognitive Screen-Plus, the objective cognitive profile of Post-COVID-19 Syndrome (PCS), as investigated by Kozik et al., reveals marked deficits in delayed verbal memory, attention, and executive functioning compared to healthy controls. Between 10% to 20% of post-COVID patients perform drastically lower than control means (exceeding 1.5 standard deviations) across affected domains. Delayed verbal memory deficits emerge as particularly severe, heavily influencing a patient’s subjective sense of impairment. Interestingly, while acute hospitalization severity, advanced age, and persistent fatigue serve as predictors of long-term attention and memory deficits, the time elapsed since initial infection, comorbid depression, and pre-existing medical conditions do not predict these cognitive drops.

## The psychological burden and vulnerable populations

The neuropsychiatric fallout of COVID-19 encompasses both primary neurocognitive deficits and severe secondary psychological morbidity in individuals who recovered from COVID-19. A nationwide, prospective cohort assessment by Saito et al., tracking individuals at 3, 6, and 12 months post-diagnosis, revealed elevated rates of psychiatric distress. Three months following acute infection, 20.1% of patients exhibit clinical anxiety, 23.6% experience depression, and 35.3% manifest high levels of COVID-19-related fear.

These elevated scores are significantly associated with specific demographics, appearing more frequently in younger individuals, females, and those who experienced mild initial acute illness. Crucially, elevated psychological scores correspond to heightened physical long COVID somatic symptoms. Granular symptom analysis shows that persistent headaches and fatigue are associated with high anxiety scores, whereas impaired concentration is associated with high depression scores. This highlights a cyclic relationship where psychological distress and somatic manifestations exacerbate one another, necessitating integrated psychiatric care.

Furthermore, the indirect psychological stress of the pandemic has impacted vulnerable pediatric populations unevenly, especially those with pre-existing neurodevelopmental conditions. Comparative cross-sectional analyses by Cohen et al. between healthy children and those with Neurofibromatosis Type 1 (NF1), a hereditary disorder characterized by cognitive and behavioral challenges, demonstrate that environmental crises exacerbate baseline vulnerabilities. Parents of children with NF1 report higher rates of generalized anxiety, social phobia, and significantly associated separation anxiety disorder during the pandemic compared to healthy peers. While typically developing children may have experienced stressors linked to remote learning and social distancing, resulting in somatization, children with NF1 suffered from healthcare access disruptions and intensified social isolation, underlining the critical need for targeted screening and tailored psychiatric interventions during public health crises.

## Conclusion

The articles collected in this Research Topic demonstrate that neuropsychiatric outcomes in COVID-19 are driven by a combination of structural brain remodeling, sex-specific cortical changes, neuroimmune loop disruptions, and profound psychological stress. Moving forward, clinical practices shall transition away from isolated treatment models. By combining rigorous cognitive scales, multi-modal neuroimaging, biomarker screenings, and integrated therapeutic strategies, we can successfully mitigate the long-term cognitive and psychiatric burden of this post-viral illness.

